# Chidamide in Combination With DCAG With or Without Venetoclax for Relapsed/Refractory Acute Myeloid Leukemia

**DOI:** 10.1002/cam4.70734

**Published:** 2025-03-10

**Authors:** Qingyang Liu, Xiawei Zhang, Lei Lv, Linming Xu, Yu Jing, Wenjing Gao, Lili Wang, Liping Dou

**Affiliations:** ^1^ State Key Laboratory of Experimental Hematology, Senior Department of Hematology The Fifth Medical Center of Chinese PLA General Hospital Beijing China; ^2^ Medical School of Chinese PLA Beijing China

**Keywords:** acute myeloid leukemia, chemotherapy, conventional, induction therapy, Venetoclax

## Abstract

**Introduction:**

Currently, there are only a few avaailable treatment options for patients with relapsed and refractory acute myeloid leukemia (R/R AML).

**Methods:**

We conducted a single‐center, phase 1 prospective study (ChiCTR2200065634) to evaluate the efficacy and safety of chidamide, demethylating drugs (azacitidine), cytarabine, aclacinomycin, and G‐CSF plus venetoclax (CDCAG‐VEN) in patients with R/R AML. The previous CDCAG regimen was used as a historical control to compare its efficacy and safety. Thirty and 22 patients received one course of CDCAG with or without a 14‐day course of venetoclax, respectively.

**Results:**

The overall response rate (ORR) was significantly higher in the CDCAG‐VEN group than in the CDCAG‐treated group (78.6% vs. 45.5%; *p* = 0.015), and the CDCAG‐VEN group achieved a better trend of measurable residual disease‐negative response (61.1% vs. 22.2%, *p* = 0.134). Compared with the CDCAG group, the CDCAG‐VEN group exhibited significantly better 1‐year overall survival (63.3% vs. 35.1%, *p* = 0.005) and progression‐free survival (76.7% vs. 36.0%, *p* = 0.022). The duration of response was notably better in the CDCAG‐VEN group than in the CDCAG group (71.2% vs. 34.3%, *p* = 0.021) and had a lower cumulative incidence of relapse (22.2% vs. 48.9%, *p* = 0.095). The neutrophil and platelet recovery times were similar between the CDCAG‐VEN and CDCAG groups (neutrophil: 18 days vs. 19 days, *p* = 0.293; platelet: 18 days vs. 19 days, *p* = 0.311). The frequencies of adverse events were comparable between both groups, except for a lower incidence of thrombosis in the CDCAG‐VEN group (0% vs. 22.7%, *p* = 0.006).

**Discussion:**

In conclusion, venetoclax in combination with CDCAG is an effective and safe treatment regimen for R/R AML, thereby rapidly identifying chemosensitive patients and inducing measurable residual disease‐negative remission in a high proportion of patients with R/R AML.

## 1 Introduction

Acute myeloid leukemia (AML) is a highly heterogeneous hematopoietic malignancy characterized by molecular abnormalities and clinical outcomes [1]. Over 50% of patients with acute myeloid leukemia (AML) experience relapse within 3 years, and the 1‐year survival rate after the first recurrence is approximately 10% [2, 3]. After two rounds of intensive induction therapy, primary refractory disease occurs in 10%–20% of younger patients with acute myeloid leukemia and 50% of older patients [4, 5]. Despite efficacy validations of a wide variety of regimens, a uniform treatment strategy for the optimal treatment of patients with relapsed/refractory acute myeloid leukemia (R/R AML) is still lacking [6]. Therefore, it is critical to develop innovative treatment strategies to improve the survival and quality of life of these patients.

A growing body of research has shown that epigenetic modifications play a crucial role in the development of chemoresistance. DNA methylation and histone acetylation are the most common epigenetic changes and can be pharmacologically reversed by DNA methyltransferase (DNMT) inhibitors or histone deacetylase (HDAC) inhibitors. Evidence indicates that demethylating drugs such as decitabine and azacitidine can inhibit DNA methyltransferase (DNMT) and reverse DNA methylation. As a result, these drugs can enhance chemosensitivity in leukemic cells and provide further rationale for a new medical approach [7]. Previous studies have indicated that histone deacetylase (HDAC) plays a crucial role in regulating chromatin structure and is frequently dysregulated in cancer, making it a promising therapeutic target for cancer treatment. Accumulating evidence from both laboratory experiments and clinical investigations suggests that HDAC inhibitors, such as chidamide, can enhance the sensitivity of cancer cells to doxorubicin treatment [8]. In our previous study, 27 patients with R/R AML with anthracycline resistance consecutively received a continuous regimen based on anthracycline combined with chidamide and decitabine. Of the 27 patients who received one course of salvage therapy, 13 achieved a complete response and one achieved a partial response. We also found that the HDAC3‐AKT‐P21‐CDK2 signaling pathway was significantly upregulated in anthracycline‐resistant AML cells compared with that in non‐resistant cells. Patients with AML with high HDAC3 levels have lower event‐free survival (EFS) and overall survival (OS) [9]. Thus, it is reasonable to use a chemotherapy regimen containing chidamide as reinduction therapy for R/R AML.

B‐cell lymphoma 2 (BCL‐2) family proteins play an important role in the intracellular apoptotic response. Venetoclax, a selective small‐molecule BCL‐2 inhibitor that binds directly to the BH3 domain of the BCL‐2 family protein, stimulates changes in mitochondrial outer membrane permeability and caspase activation to achieve rapid apoptosis of tumor cells [10]. Venetoclax has been approved for the treatment of AML in the elderly or those who cannot tolerate intensive chemotherapy [11]. In a study of venetoclax monotherapy in patients with R/R AML, the overall response rate (ORR) was only 19%, and the median progression‐free survival (PFS) was very short [12]. Venetoclax cannot precisely attack tumor cells but enhances the anti‐tumor effect of anthracyclines through apoptosis. Therefore, in AML, it is crucial to combine venetoclax with other drugs [13]. A phase II study of the VAA regimen (venetoclax plus cytarabine and azacitidine) in 30 patients with R/R AML reported a combined complete remission (CR) and complete remission with incomplete hematologic recovery (CRi) rate of 63.3% and a median duration of response of 18.3 months. Notably, 14 (73.7%) of the 19 patients who achieved a CRc showed undetectable measurable residual disease by flow cytometry [14]. In the current study, we designed a regimen that included chidamide, demethylating drugs (azacitidine), cytarabine, aclacinomycin, G‐CSF, and venetoclax (CDCAG‐VEN) for the treatment of patients with R/R AML.

We assessed the activity and safety of the CDCAG‐VEN regimen in patients with R/R AML. The previous regimen, including chidamide, demethylating drugs (decitabine), cytarabine, aclacinomycin, and G‐CSF (CDCAG), was used as a historical control to compare the efficacy and safety of the CDCAG‐VEN regimen.

## 2 Materials and Methods

### 2.1 Patients

We conducted a single‐center, phase 1 prospective study to evaluate the efficacy and safety of the CDCAG‐VEN regimen in patients with R/R AML, excluding cases of acute promyelocytic leukemia. The trial is registered in the Chinese Clinical Trial Registry ChiCTR2200065634. All patients were treated at the Chinese PLA General Hospital between January 12, 2022 and December 20, 2023. Patients with R/R AML (excluding acute promyelocytic leukemia cases) who received the CDCAG regimen at the Chinese PLA General Hospital from June 30, 2016 to December 09, 2017, were retrospectively analyzed.

This study was conducted in accordance with the Declaration of Helsinki and was approved by the Institutional Review Board of each participating institution. All patients who were enrolled in the study provided written informed consent.

Patients were required to have an Eastern Cooperative Oncology Group (ECOG) performance status ranging from 0 to 2, as well as demonstrate sufficient cardiac, liver, and renal function. Refractory AML is defined as failure to achieve CR after one or more cycles of standard induction chemotherapy. Relapsed AML was defined as the recurrence of blasts in the peripheral blood (PB), BM blasts ≥ 5%, or the development of extramedullary disease. Detailed information about the inclusion and exclusion criteria is presented in Table S1.

### 2.2 Treatments

All patients in this study were treated with the CDCAG‐VEN regimen over a 28‐day cycle: chidamide 30 mg was administered orally on days 1, 4, 8, and 11; azacitidine 75 mg/m^2^ was administered subcutaneously on days 1–7; cytarabine: 75–100 mg/m^2^ bid was administered intravenously on days 1–5; aclacinomycin 10 mg/m^2^/day was administered on days 1, 3, and 5; and G‐CSF 5 μg/day was administered until white blood cell (WBC) > 20 × 10^9^/L. Venetoclax was administered orally on days 1–14 (100 mg for day 1, 200 mg for day 2, 400 mg for days 3–14, Figure S1). During treatment, symptomatic and supportive therapies were administered to protect liver and kidney function (hydration, alkalinization), while preventing tumor lysis syndrome and infection. If patients need to initiate antifungal treatment or other moderate or strong CYP3A4 inhibitors, the venetoclax dose should be adjusted according to the recommended prescription information.

Patients received the CDCAG regimen over a 28‐day cycle: chidamide 30 mg was administered orally on days 1, 4, 8, and 11; decitabine 20 mg/m^2^ was administered intravenously on days 1–5; cytarabine 50 mg/m^2^ was administered on days 1–7 if WBC ≥ 20 × 10^9^/L and days 3–7 if WBC < 20 × 10^9^/L; aclacinomycin 10 mg/m^2^ was administered on days 3–7; and G‐CSF 5 μg/day was administered until WBC > 20 × 10^9^/L (Figure S1).

The bone marrow was evaluated on day 28 after the start of therapy. Measurable residual disease (MRD) by flow cytometry during each treatment cycle. Samples with at least 500,000 cells were considered evaluable. The MRD was evaluated with WBC, defining the minimal number of events to call MRD positivity as 50. The MRD was evaluated with a sensitivity of 0.01%–0.10%, defining MRD negativity as < 0.1% and MRD positivity as ≥ 0.1%. Baseline and scheduled samples were collected for genomic sequencing. Cardiac function assessments, as well as regular tests, such as biochemistry, electrocardiography, and echocardiography, monitored cardiac, renal, and hepatic functions. Additional tests were performed at the physician's discretion. Adverse events were assessed according to standard criteria, with continuous monitoring throughout the study.

Patients who are eligible for hematopoietic stem cell transplantation are recommended to undergo hematopoietic stem cell transplantation as soon as possible after complete remission. If partial remission (PR) is achieved, another cycle of treatment is performed to assess remission. If the symptoms do not resolve, the patient should be immediately switched to another treatment option.

### 2.3 Endpoints

The primary endpoint was the overall response rate after one cycle of induction therapy (ORR: complete remission plus remission with incomplete hematologic recovery plus partial response). The secondary endpoints were composite complete remission (CRc, CR + CRi), PR, MRD, OS, PFS, duration of response (DOR), cumulative incidence of relapse (CIR), and treatment‐related adverse events after one cycle of induction.

CR was defined as bone marrow with less than 5% blasts, a platelet count > 100,000/mm^3^, red‐cell transfusion independence, and an absolute neutrophil count > 1000 cells/mm^3^. CRi was defined as all of the criteria for CR, except for neutropenia (absolute neutrophil count, ≤ 1000/mm^3^) or thrombocytopenia (platelet count, ≤ 100,000/mm^3^). PR was defined for all hematologic criteria of CR, decreased bone marrow blast percentage to 5%–20%, and decreased pre‐treatment bone marrow blast percentage by at least 50%. OS was defined as the time interval from treatment initiation to the date of death from any cause. PFS was defined as the time from enrollment to the occurrence of any event, including disease progression, cessation of treatment for any reason, or death. DOR was defined among responders as the duration between the date of response and the date of disease relapse or death from any cause, whichever occurred first. CIR was defined as the incidence of relapse after CRc, with death in CRc as a competing risk. Treatment‐related adverse events were defined as events that occurred from the first dose of the study treatment to 30 days after treatment discontinuation. The severity of adverse events was graded according to the National Cancer Institute Common Terminology Criteria for Adverse Events, version 5.0 [15].

### 2.4 Data Collection

Relevant data of patients were collected through inpatient and outpatient medical record systems and telephone follow‐up, mainly including the age of onset, sex, physical condition at the first diagnosis, blood routine, biochemistry, original bone marrow cell count at diagnosis, bone marrow assay after the first admission, leukemia immunophenotyping, karyotype, fusion gene, other medical history, previous chemotherapy regimen, drug dosage, French‐American‐British (FAB) classification, chest and abdominal examination (including CT or color Doppler ultrasound), treatment history, survival time, and recurrence.

### 2.5 Sample Size

The sample size was calculated according to the primary endpoint (ORR) of the study. In a study conducted with a Chinese population, the ORR after induction therapy with the “CDCAG” regimen was reported to be 46.2% [16]. The ORR of venetoclax along with intensive chemotherapy for patients with R/R AML was 75%–78.1% [17, 18]. Therefore, in the sample calculation, we selected 78.1% as the reference ratio and determined the expected ORR for patients. This study was planned at a one‐sided significance level α = 5% with a power of 1‐β = 80%. Twenty‐six patients were included in the CDCAG‐VEN group. Allowing for a drop‐out rate of 10%, 30 patients were required.

### 2.6 Statistics

Continuous data are described using median values and ranges (minimum‐maximum). Categorical data are described as *n* (%). The ORR, CR, CRi, CRc, and MRD‐negative rates were calculated using 95% confidence intervals (CIs). Fisher's exact test or chi‐squared test was used to compare the rates between groups. The Kaplan–Meier method was used to estimate the DOR, PFS, and OS. The cumulative incidence of relapse was estimated using a competing risk model. Death without relapse was defined as a competing event for relapse. Safety analyses were performed for all patients who received at least one cycle of CDCAG‐VEN or CDCAG chemotherapy. Any difference for which two‐sided *p* < 0.05 was considered statistically significant. The statistical analyses were performed using the statistical software environment R (version 4.1.2), SPSS (version 22.0), and GraphPad Prism (version 7.0).

## 3 Results

### 3.1 Patients

From January 12, 2022 to December 20, 2023, 30 patients with R/R AML were enrolled. Thirty patients received the CDCAG‐VEN regimen, and 22 received the CDCAG regimen. The baseline characteristics were similarly distributed between the CDCAG‐VEN and CDCAG groups (Table 1). Similar proportions of patients in the CDCAG‐VEN and CDCAG groups had refractory AML (53.3% vs. 68.2%) or relapsed AML (46.7% vs. 31.8%). The percentage of bone marrow blasts in R/R patients before chemotherapy was similar between the CDCAG‐VEN‐ and CDCAG‐treated patients (52% vs. 77%, *p =* 0.104). The number of prior chemotherapy cycles was comparable between the CDCAG‐VEN‐ and CDCAG‐treated patients (*p =* 0.675). In addition, the number of patients with prior Stem Cell Transplantation (SCT) was one of 30 (3.3%) in the CDCAG‐VEN group and one of 22 (5.0%) in the CDCAG group. Four patients (13.3%) had complex karyotypes in the CDCAG‐VEN group and two patients (9.1%) in the CDCAG group (*p =* 0.636). The mutation frequency was similarly distributed between CDCAG‐VEN and CDCAG patients, except for the higher frequency of TP53 (18.2% vs. 0%, *p =* 0.071) and a lower frequency of NPM1 (4.5% vs. 25.0%, *p =* 0.066) mutations in the CDCAG‐VEN group (Figures S2). Comparing the baseline characteristics between the two groups, the differences were not significant. After CDCAG‐VEN chemotherapy, 14 patients transitioned to allo‐HSCT. By contrast, in the CDCAG group, four patients were bridged to allo‐HSCT (46.7% vs. 18.1%, *p =* 0.097).

**TABLE 1:** Demographics, baseline, and treatment characteristics of CDCAG‐VEN‐ and CDCAG‐treated patients.

Patient characteristics	CDCAG‐VEN (*n* = 30)	CDCAG (*n* = 22)	*p*
Age, years, median (range)	44 (15–74)	41 (12–71)	0.254
Sex, *n* (%)			0.516
Male	15 (50.0)	13 (59.1)	
Disease status, *n* (%)			0.281
Refractory	16 (53.3)	15 (68.2)	
Relapse	14 (46.7)	7 (31.8)	
ECOG performance status, *n* (%)			0.680
0–1	16 (53.3)	13 (59.1)	
2	14 (46.7)	9 (40.9)	
Bone marrow blasts, median% (range)	52 (13–95)	77 (45–95)	0.104
NCCN 2023 risk group at newly diagnosed, *n* (%)*			0.116
Favorable	3 (10.0)	3 (13.6)	
Intermediate	15 (50.0)	16 (72.7)	
Adverse	12 (40.0)	3 (13.6)	
No. of prior therapies, median (range)	2 (1–12)	2 (1–16)	0.489
Prior therapies, *n* (%)			0.675
HMA only	18 (60.0)	8 (36.3)	
HMA and chidamide	1 (3.3)	0	
SCT	1 (3.3)	1 (5.0)	
Bridging allo‐HSCT, *n* (%)	14 (46.7)	4 (18.1)	0.097
Karyotype, *n* (%)			0.636
Normal karyotype	26 86.7)	20 (90.9)	
Complex karyotype*	4 (13.3)	2 (9.1)	
Mutations, *n* (%)	*n* = 22	*n* = 16	
*DNMT3A*	6 (27.3)	4 (25.0)	0.875
*NPM1*	1 (4.5)	4 (25.0)	0.066
*SRSF2*	2 (9.1)	0	0.215
*FLT3‐ITD*	4 (18.2)	6 (37.5)	0.182
*IDH1*	4 (18.2)	3 (18.6)	0.964
*IDH2*	3 (13.6)	2 (12.5)	0.919
*RUNX1*	2 (9.1)	2 (12.5)	0.735
*K/NRAS*	6 (27.3)	4 (25.0)	0.875
*TP53*	4 (18.2)	0	0.071

Abbreviations: Allo‐HSCT, allogeneic hematopoietic stem cell transplantation; AML, acute myeloid leukemia; CDCAG, chidamide, demethylating drugs (decitabine), cytarabine, aclacinomycin, and G‐CSF; CDCAG‐VEN, chidamide, demethylating drugs (azacitidine), cytarabine, aclacinomycin, G‐CSF, and venetoclax; ECOG, Eastern Cooperative Oncology Group; HMA, hypomethylating agents; NCCN, National Comprehensive Cancer Network; SCT, stem cell transplantation.

* Complex karyotype is defined as ≥ 3 clonal chromosomal abnormalities.

### 3.2 Clinical Responses

**FIGURE 1:**
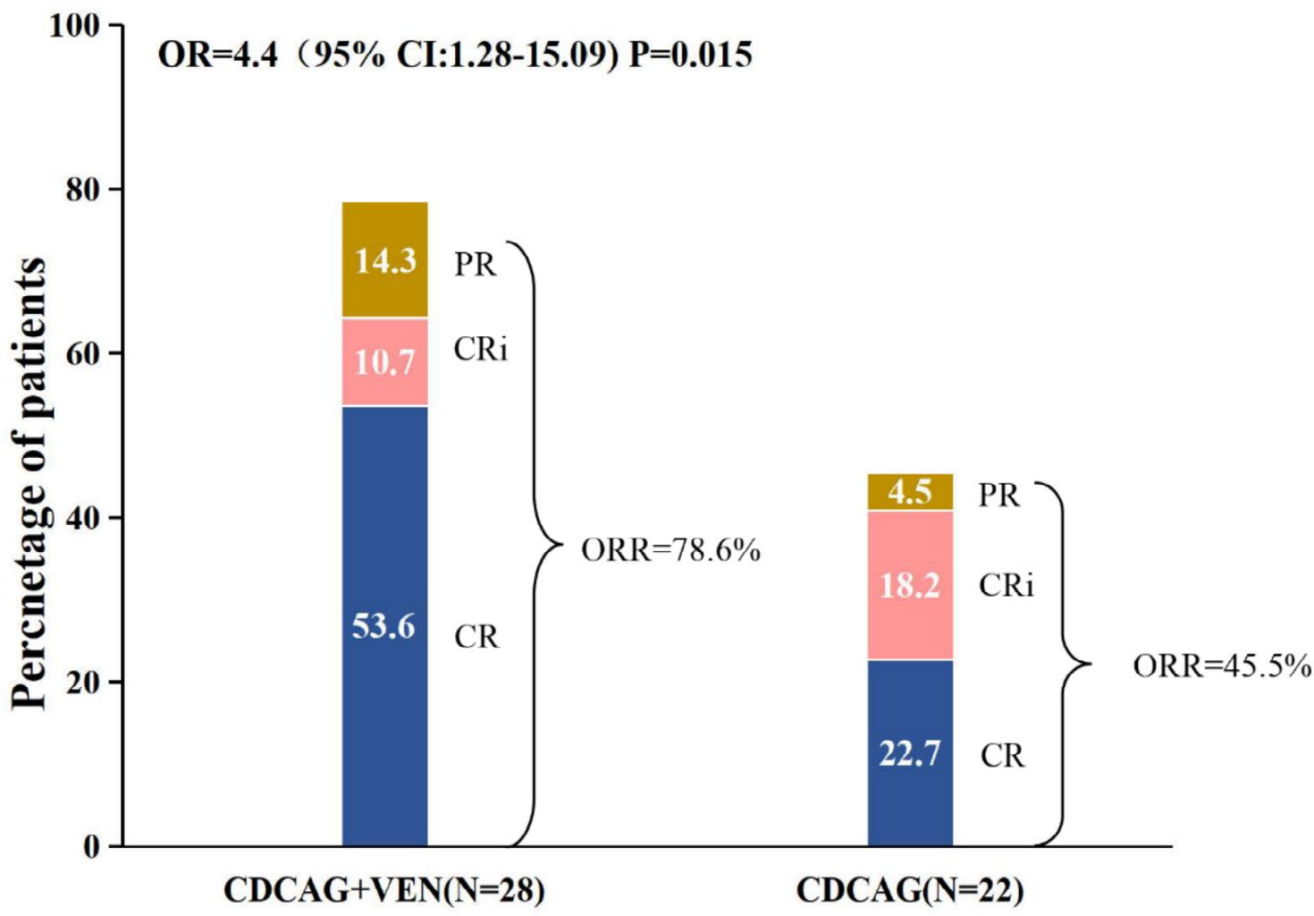
Frequency of complete remission, complete remission with incomplete hematological recovery, partial remission, and overall response rate in CDCAG‐VEN‐ (*n* = 28) and CDCAG‐ (*n* = 22) treated patients. CDCAG, chidamide, demethylating drugs (decitabine), cytarabine, aclacinomycin, and G‐CSF; CDCAG‐VEN, chidamide, demethylating drugs (azacitidine), cytarabine, aclacinomycin, G‐CSF, and venetoclax; CI, confidence interval; CR, complete remission; CRi, complete remission with incomplete hematological recovery; ORR, overall response rate; PR, partial remission.

All 30 patients received one cycle of CDCAG‐VEN chemotherapy, and two patients refused bone marrow puncture after the first course of treatment. The ORR was 78.6% (22/28, 95% CI: 58.4–91.0) in the CDCAG‐VEN group and 45.5% (10/22, 95% CI: 25.1–67.3) in the CDCAG group (*p =* 0.015). Fifteen patients (53.6%; 95% CI: 34.2–72.0) achieved a CR, three patients (10.7%; 95% CI: 2.8–29.4) achieved a CRi, and four patients (14.3%; 95% CI: 4.7–33.6) achieved a PR in the CDCAG‐VEN group. Five patients (22.7%; 95% CI: 8.7–45.8) achieved a CR, four patients (18.2%; 95% CI: 6.0–41.0) achieved a CRi, and one patient (4.6%; 95% CI: 0.2–24.9) achieved a PR in the CDCAG group. The CRc was 64.3% (18/22, 95% CI: 44.1–80.7) in the CDCAG‐VEN group and 40.9% (9/22, 95% CI: 21.5–63.3) in the CDCAG group. After one treatment cycle, 11 patients in the CDCAG‐VEN group and only two patients in the CDCAG group achieved a CRc with MRD negative status (CDCAG‐VEN 61.1% and CDCAG 22.2%, *p =* 0.134, Table 2, Figure 1). Furthermore, for patients with National Comprehensive Cancer Network (NCCN)‐associated adverse risk, the ORR in the CDCAG‐VEN group was 70.0% (7/10, 95% CI: 35.4–91.9) and that in the CDCAG group was 33.3% (1/3, 95% CI: 1.8–87.5, *p =* 0.252). The CRc was 50.0% (5/10, 95% CI: 20.1–79.9) and 33.3% (1/3, 95% CI: 1.8–87.5) in the CDCAG‐VEN and CDCAG groups, respectively (*p =* 0.612, Table S4).

**TABLE 2:** Treatment response after one cycle of CDCAG–VEN and CDCAG treatment.

Outcomes	CDCAG‐VEN (*n* = 28)	CDCAG (*n* = 22)	OR (95% CI)	*p*
ORR, *n* (%, 95% CI)	78.6 (58.4–91.0)	45.5 (25.1–67.3)	4.40 (1.28–15.09)	0.015
CRc, *n* (%, 95% CI)	64.3 (44.1–80.7)	40.9 (21.5–63.3)	2.60 (0.82–8.20)	0.100
CR	53.6 (34.2–72.0)	22.7 (8.7–45.8)		
CRi	10.7 (2.8–29.4)	18.2 (6.0–41.0)		
MRD–CRc, *n* (%, 95% CI)	61.1 (36.1–81.7)	22.2 (4.0–59.8)	6.47 (1.26–33.34)	0.134
PR, *n* (%, 95% CI)	14.3 (4.7–33.6)	4.6 (0.2–24.9)	3.50 (0.36–33.82)	0.254
NR, *n* (%, 95% CI)	21.4 (9.0–41.5)	54.6 (32.7–74.9)	0.23 (0.07–0.78)	0.015

Abbreviations: CDCAG, chidamide, demethylating drugs (decitabine), cytarabine, aclacinomycin, and G‐CSF; CDCAG‐VEN, chidamide, demethylating drugs (azacitidine), cytarabine, aclacinomycin, G‐CSF, and venetoclax; CI, confidence interval; CR, complete remission; CRc, composite complete remission; CRi, complete remission with incomplete hematological recovery; MRD, measurable residual disease; NR, no response; OR, odds ratio; ORR, overall response rate; PR, partial remission.

**FIGURE 2:**
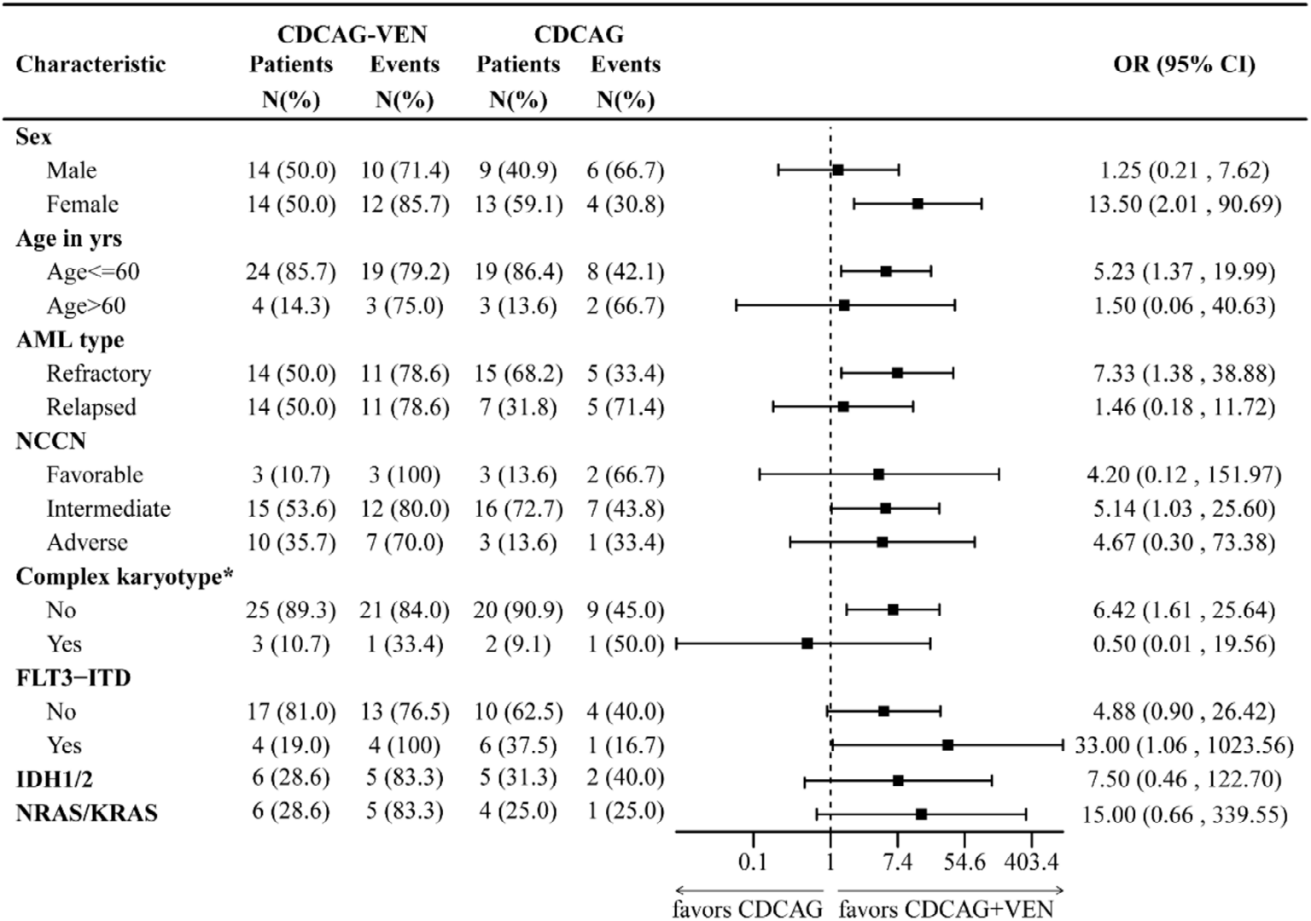
Impact of patient and disease characteristics on achieving overall response remission in CDCAG‐VEN and CDCAG patients. CDCAG, chidamide, demethylating drugs (decitabine), cytarabine, aclacinomycin, G‐CSF; CDCAG‐VEN, chidamide, demethylating drugs (azacitidine), cytarabine, aclacinomycin, G‐CSF, and venetoclax; CI, confidence interval; NCCN, National Comprehensive Cancer Network; OR, odds ratio. *Complex karyotype is defined as ≥ 3 clonal chromosomal abnormalities.

Exploratory analysis of response in clinical and genetic subgroups showed a high consistency of a better response to CDCAG‐VEN compared with CDCAG across most subgroups, notably in patients with FLT3::ITD, IDH1/2, and NRAS/KRAS mutations, whereas the benefit was less pronounced in patients aged > 60 years and with a complex karyotype (Figure 2).

### 3.3 Survival

**FIGURE 3:**
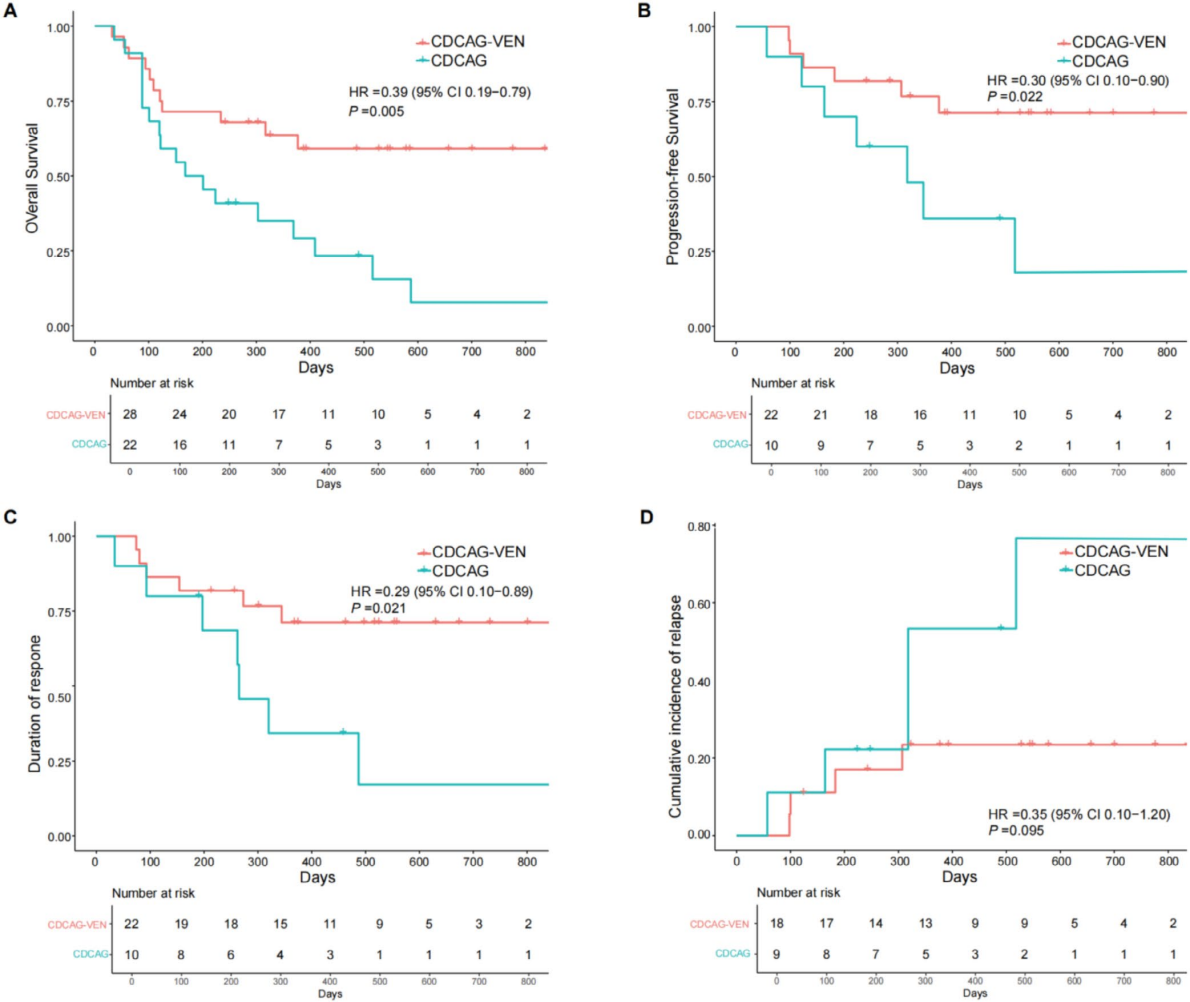
Survival outcomes in CDCAG‐VEN‐ and CDCAG‐treated patients. (A) Overall survival (OS) in CDCAG‐VEN‐ and CDCAG‐treated patients. (B) Progression‐free survival (PFS) in CDCAG‐VEN‐ and CDCAG‐treated patients. (C) Duration of response (DOR) in CDCAG‐VEN‐ and CDCAG‐treated patients. (D) Cumulative incidence of relapse (CIR) in CDCAG‐VEN‐ and CDCAG‐treated patients. CDCAG, chidamide, demethylating drugs (decitabine), cytarabine, aclacinomycin, and G‐CSF; CDCAG‐VEN, chidamide, demethylating drugs (azacitidine), cytarabine, aclacinomycin, G‐CSF, and venetoclax.

The median follow‐up was 544 (243–917) d in the CDCAG‐VEN group and 490 (248–2203) d in the CDCAG group. No patients experienced early death within 30 days of therapy initiation. Three patients died from relapse of AML in the CDCAG‐VEN group, and four patients died from relapse of AML in the CDCAG group. The 1‐year OS was 63.6% in the CDCAG‐VEN group compared with 35.1% in the CDCAG group (HR: 0.39, 95% CI: 0.19–0.79; *p =* 0.005, Figure 3A), and the 1‐year PFS was 76.7% in the CDCAG‐VEN group compared with 15.9% in the CDCAG group (HR: 0.30, 95% CI: 0.10–0.90; *p =* 0.022, Figure 3B). The 1‐year DOR was significantly higher in the CDCAG‐VEN group than in the CDCAG group (71.2% vs. 34.3%; HR: 0.29, 95%, 0.10–0.89; *p =* 0.021, Figure 3C). The cumulative incidence of relapse at 1 year was 22.2% in the CDCAG‐VEN group and 48.9% in the CDCAG group (HR: 0.35, 95%, 0.10–1.20; *p =* 0.095, Figure 3D, Table S5).

For patients achieving CRc after one cycle of CDCAG‐VEN, 11 and seven patients achieved MRD‐negative and MRD‐positive status, respectively. The 1‐year OS of MRD‐negative patients was 81.8%, and the 1‐year OS of MRD‐positive patients was 64.3% (HR: 0.78, 95% CI: 0.13–4.88; *p =* 0.794, Figure S3A). The 1‐year PFS was 81.8% compared with 53.6%, respectively (HR: 0.51, 95% CI: 0.10–2.62; *p =* 0.415, Figure S3B). Two patients achieved CRc with MRD‐negative status after one cycle of CDCAG. One patient relapsed at 318 days and died at 516 days, whereas the other patient survived long‐term. Among the 28 patients treated with CDCAG‐VEN, those who received allo‐HSCT showed improvements in OS compared with patients who did not receive allo‐HSCT (OS at 1 year: 80.9% vs. 36.4%, *p =* 0.002, Figure S4A). Compared with patients who did not receive allo‐HSCT, the CDCAG+VEN group showed a trend of increased OS at 1 year compared with the CDCAG group, albeit with non‐significant statistical differences between the groups (36.4% vs. 25.0%, *p* = 0.606, Figure S4B).

### 3.4 Safety

The most commonly observed treatment‐related toxicities of any grade were hematological adverse events (AE) reported in all patients. After one cycle of CDCAG‐VEN induction therapy, the most common nonhematological adverse event was hypokalemia (22/30, 73.3%). Non‐hematological AE of grade 3 or 4 included pneumonia (5/30, 16.7%), hypokalemia (5/30, 16.7%), hyponatremia (4/30, 13.3), and hypocalcemia (1/30, 3.3%). Among the patients treated with the CDCAG‐VEN regimen, ten (33.3%) developed pulmonary infection, five (16.7%) of whom developed grade 3/4 pneumonia, and one died of infectious shock on the 19th day after chemotherapy. Among the patients treated with CDCAG, ten (45.5%) developed pneumonia, seven (31.8%) of whom developed grade 3/4 pneumonia. The CDCAG group included five patients with thrombosis, whereas the CDCAG‐VEN group contained no cases of thrombosis (*p =* 0.006, Table 3).

**TABLE 3:** Treatment‐emergent adverse events of CDCAG‐VEN and CDCAG‐treated patients.

Event	CDCAG‐VEN (*n* = 30)		CDCAG (*n* = 22)		*p*
	Any grade (%)	Grade 3/4 (%)	Any grade (%)	Grade 3/4 (%)	
Pneumonia	10 (33.3)	5 (16.7)	10 (45.5)	7 (31.8)	0.375
Abnormal ALT/AST	14 (46.7)	0	9 (40.9)	0	0.680
Nausea/vomiting	2 (6.7)	0	1 (4.5)	0	0.746
Diarrhea	4 (13.3)	0	1 (4.5)	0	0.288
Oral ulcer	1 (3.3)	0	1 (4.5)	0	0.822
Urinary tract hemorrhage	3 (10.0)	0	0	0	0.127
Drug‐induced kidney injury	2 (6.7)	0	1 (4.5)	0	0.746
Hypoalbuminemia	12 (40.0)	0	16 (72.7)	0	0.301
Hyponatremia	7 (23.3)	4 (13.3)	1 (4.5)	1 (4.5)	0.064
Hypokalemia	22 (73.3)	5 (16.7)	19 (86.4)	5 (22.7)	0.256
Hypocalcemia	16 (53.3)	1 (3.3)	14 (63.6)	0	0.458
Thrombosis	0	0	5 (22.7)	0	0.006

Abbreviations: CDCAG, Chidamide, demethylating drugs (decitabine), cytarabine, aclacinomycin, and G‐CSF; CDCAG‐VEN, Chidamide, demethylating drugs (azacitidine), cytarabine, aclacinomycin, G‐CSF, and venetoclax.

The median times to neutrophil recovery (> 0.5 × 10^9^/L) were 18 and 19 days in the responding CDCAG‐VEN‐treated and CDCAG patients (*p =* 0.293) from day 1 of chemotherapy, respectively. The median time to platelet recovery (20 × 10^9^/L) was 18 and 19 days in the CDCAG‐VEN‐treated and CDCAG patients (*p =* 0.311), respectively. The CDCAG‐VEN‐treated and CDCAG patients received packed red cell transfusions of 9.5 and 10.2 units (*p =* 0.282) and had a platelet transfusion rate of 9.2 and 10.0 units, respectively (*p =* 0.322). Thus, the neutrophil count and platelet recovery times were comparable between the CDCAG‐VEN and CDCAG regimens (Table S6).

## 4 Discussion

In this single‐arm, phase 1 trial, we aimed to show that the addition of venetoclax to the CDCAG regimen was well tolerated and resulted in higher response rates than the CDCAG regimen. High response rates were observed in patients across NCCN and mutation subgroups. The CDCAG‐VEN regimen not only was associated with deep remission, with a high rate of MRD‐negative remission among patients receiving chemotherapy, but also showed encouraging safety results.

At present, there is no standardized treatment protocol for R/R AML, highlighting an urgent need for effective treatment options to increase remission rates and improve prognosis. An ORR of 45.5%, including a CRc rate of 40.9%, was obtained after one cycle in the CDCAG cohort. The response rate in the CDCAG‐treated cohort was consistent with previously reported response rates for this regimen [16, 19]. However, low ORR and CRc rates were observed in patients at adverse risk (ORR: 33.3%, CRc: 33.3%). The rate of deep remission with the CDCAG regimen was also limited (MRD‐CRc: 22.2%). By contrast, the ORR of 28 patients after one cycle of CDCAG‐VEN treatment was 78.6%, including a CRc rate of 64.9%. We observed the ORR and CRc across NCCN risk groups (favorable: 100.0% [ORR], 100.0% [CRc]; intermediate: 80.0% [ORR], 66.7% [CRc]; adverse: 70.0% [ORR], 50.0% [CRc]), as well as a high MRD‐negative rate (MRD‐CRc: 61.1%). The results of survival analyses suggest that response to CDCAG‐VEN significantly improved the survival rate of patients. The CDCAG‐VEN group demonstrated significantly improved 1‐year OS (63.6% vs. 35.1%, *p =* 0.005) and PFS (76.7% vs. 15.9%, *p =* 0.022) compared with the CDCAG group. We also observed that allo‐HSCT could benefit patients in the CDCAG‐VEN group in terms of OS and PFS compared with the CDCAG group. Our findings demonstrate that CDCAG‐VEN is an effective intensive treatment option for patients with R/R AML, particularly as a bridge to allo‐HSCT.

The promising response rate of this novel regimen was attributed to the 14‐day administration of venetoclax once daily. The BCL‐2 protein is commonly overexpressed in patients with AML, leading to chemotherapy resistance and worse prognosis. Accumulating evidence shows that venetoclax is a highly selective and effective BCL‐2 inhibitor that can restore the apoptotic potential of cancer cells [20, 21]. Venetoclax combined with low‐dose cytarabine or demethylating drugs has been approved for older patients with AML by the Food and Drug Administration. With its entire repertoire yet to be explored, venetoclax has changed the therapeutic landscape of hematological malignancies, most particularly chronic lymphocytic leukemia, AML, and multiple myeloma [22, 23]. Venetoclax monotherapy is well tolerated in patients with AML, but its efficacy is suboptimal, especially for high‐risk AML subtypes or patients with complex genomic alterations. A phase II study involving 32 patients diagnosed with R/R AML showed that six achieved a CRc, yielding an ORR of 19% [24]. Therefore, it is essential to combine venetoclax with other drugs. The addition of venetoclax to fludarabine, cytarabine, G‐CSF, and idarubicin (FLAG‐IDA) in R/R AML also translated into high response rates, including a composite complete response of 90% in newly diagnosed patients and 67% in R/R patients [25]. Overall, these findings suggest that incorporating venetoclax into conventional induction chemotherapy regimens can increase CR rates in adult patients with AML. Some studies have suggested that patients with specific gene mutation types (e.g., FLT3, TP53, K/NRAS, DNMT3A, SF3B1) also experience a significantly increased incidence of CRc when treated with a chemotherapy regimen combined with venetoclax [12, 26, 27]. With the previous CDCAG regimen, we observed that patients with FLT3::ITD had a lower ORR (16.7%), which was improved by the addition of venetoclax. The ORR of CDCAG‐VEN in patients with FLT3::ITD was 100%. In addition, the IDH1/2 mutation subgroups showed a high response in the CDCAG‐VEN group (CDCAG‐VEN: 83.3% [ORR]; CDCAG: 40.0% [ORR]). However, in the CDCAG‐VEN group, we observed a poor CRc (25%) mainly in patients carrying TP53. The integration of epigenetic modifiers might enhance resistance to VEN‐based therapy in patients with other mutations. Regarding TP53 mutations, there is still a need for additional therapeutic strategies, and the combination of TP53‐targeting agents may be the next step.

The CDCAG‐VEN regimen showed a favorable complete remission rate in the context of published studies on intensive chemotherapy in fit adults with R/R AML. A retrospective study reported that the combination of venetoclax and azacitidine was used in 39 R/R patients. The ORR in R/R patients was only 37%, including 13% CR and 8% CRi [28]. Comparing with the azacititine+venetoclax regimen, CDCAG‐VEN regimen has the better ORR (78.6% vs. 37%). Patients with R/R AML are usually treated with the FLAG‐IDA + Ven or FLA‐IDA + Ven regimens. A retrospective cohort study compared the safety and efficacy of FLA‐IDA without or with venetoclax [29]. This study reported that the ORR in the FLA‐IDA plus venetoclax (FLAVID) group was similar to our study (78.6% vs. 78%). However, the MRD‐negative rate was significantly higher in the CDCAG‐VEN regimen compared to FLAVIDA‐treated patients. This result suggested that the CDCAG‐VEN regimen can lead to a better complete remission in R/R AML patients. To further analyze the efficacy of different mutations in the two groups, we compared the FLT3‐ITD mutation and the KARS/NARS mutation. Compared with the FLAVIDA regimen, the CDCAG‐VEN regimen has the better ORR (FLT3‐ITD: 100% vs. 88%; KARS/NARS: 83.3% vs. 75%). In a study of venetoclax combined with FLAG‐IDA (FLAGVIDA) in patients with R/R AML, the ORR was 78%, which was also similar to the CDCAG‐VEN group; this may be due to the fact that the CDCAG‐VEN group had more patients with adverse risk (40% vs. 28%) [30].

Because toxicity is a major concern when adding novel drugs to existing regimens, safety and toxicity are essential outcomes of this study. Common grade 3 and 4 non‐hematological AEs occurring in the CDCAG‐VEN group were pneumonia (16.7%) and hypokalemia (16.7%). These results were similar in frequency and intensity to those reported in previous studies for venetoclax plus intensive chemotherapy [31, 32]. Venetoclax has been reported to have anti‐migration, anti‐invasion, and anti‐thrombus formation effects [33]. This is consistent with our expectations, and we observed no thrombosis in the CDCAG‐VEN group. On the other hand, because our study was a comparison with a historical cohort, with the progression in supportive care, the incidence of these adverse events will be significantly reduced.

A comparison of neutrophil and platelet count recovery revealed comparable levels between the CDCAG‐VEN and CDCAG regimens, suggesting that a 14‐day course of venetoclax does not add significant hematologic toxicity to the CDCAG regimen.

This trial has several limitations. First, only 30 patients were included, and the median follow‐up duration was relatively short. The conclusions of the subgroup analysis may be biased or overinterpreted due to the small sample size; therefore, additional studies with more patients and long‐term follow‐up are required. Second, this study defined prior ≥ 1‐course induction failure as refractory failure. Some patients were adjusted to the CDCAG‐VEN regimen after receiving one course of failed induction at the clinical stage. This might have led to an overestimation of the activity of the CDCAG‐VEN regimen. Third, the short‐term follow‐up prevented us from reaching a definitive conclusion regarding the survival benefits of the CDCAG‐VEN regimen, although the benefit in CRc could lead to an overall survival benefit, and MRD‐negative remission is widely considered a good surrogate endpoint of survival in patients with AML.

In conclusion, this study provides valuable insights into the efficacy, survival outcomes, and safety of CDCAG‐VEN compared with CDCAG in patients with R/R AML. These results support the potential of the CDCAG‐VEN regimen as a promising therapeutic approach in this challenging patient population and warrant further investigation and clinical application.

## Author Contributions

**Qingyang Liu:** conceptualization, investigation, writing – original draft, writing – review and editing, visualization, validation, methodology, software. **Xiawei Zhang:** investigation, data curation. **Lei Lv:** investigation. **Linming Xu:** investigation. **Yu Jing:** investigation. **Wenjing Gao:** investigation. **Lili Wang:** visualization, project administration, supervision, resources. **Liping Dou:** funding acquisition, formal analysis, project administration, data curation, resources, supervision, writing – review and editing.

## Ethics Statement

The study was approved by the Ethics Committee of the Chinese PLA General Hospital (S2022‐240‐01) and was executed in strict adherence to the principles outlined in the Declaration of Helsinki.

## Conflicts of Interest

The authors declare no conflicts of interest.

## Supporting information


Data S1.

Data S2.

## Data Availability

The date in this study can be requested upon reasonable use.
