# Supplementary material for: Chidamide in Combination With DCAG With or Without Venetoclax for Relapsed/Refractory Acute Myeloid Leukemia
Source: Cancer Med. 2025 Mar 10;14(5):e70734. doi: 10.1002/cam4.70734 (PMC11891779; doi:10.1002/cam4.70734)
Supplement: Supplementary file 1 — Data S1. [file CAM4-14-e70734-s002.docx]

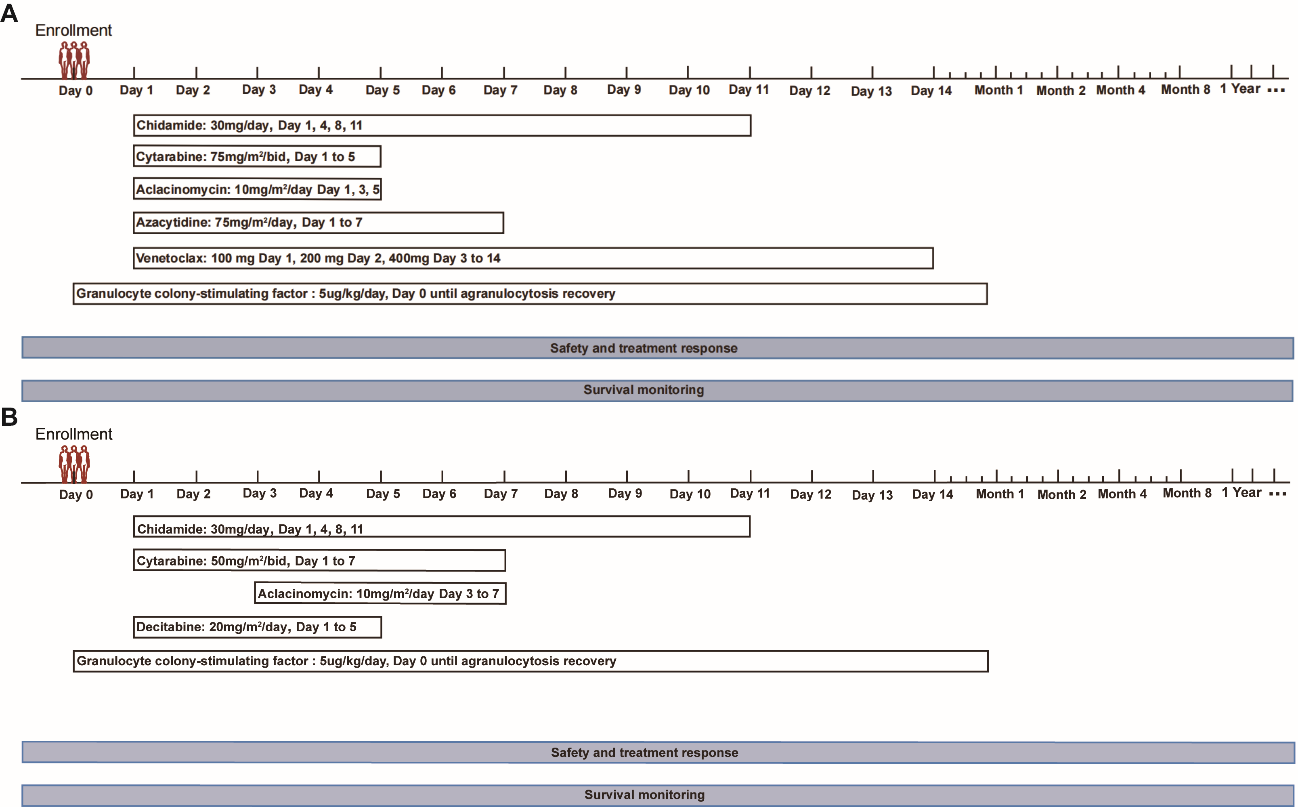


Figure S1. Treatment schema of CDCAG-VEN and CDCAG. (A) CDCAG-VEN: Chidamide, demethylating drugs (azacitidine), cytarabine, aclacinomycin, G-CSF, and venetoclax; (B) CDCAG: Chidamide, demethylating drugs (decitabine), cytarabine, aclacinomycin, and G-CSF; bid, twice a day; Day, the time after enrollment.


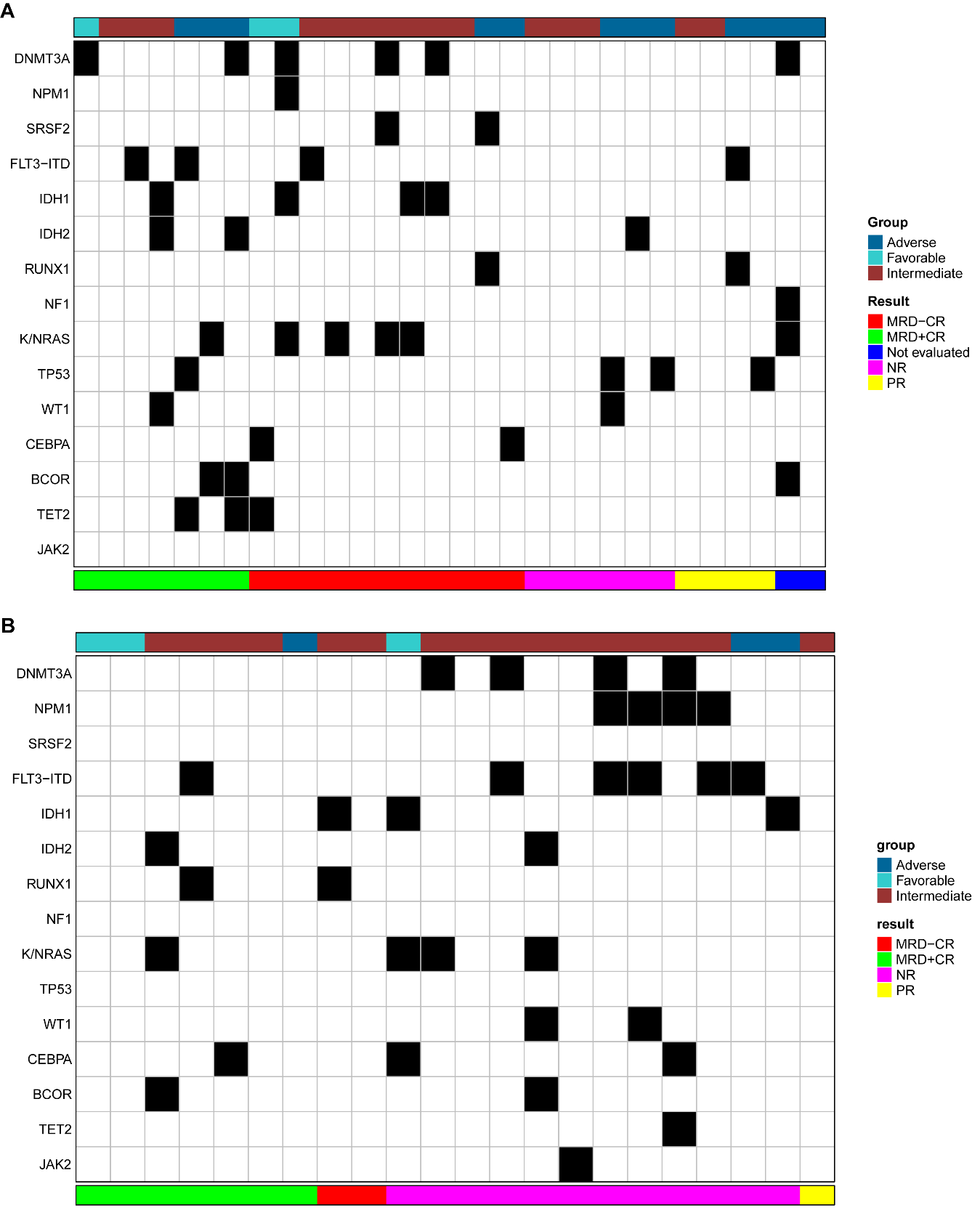


Figure S2. Genomic landscape in CDCAG-VEN patients (n = 30) and CDCAG patients (n = 22). (A) CDCAG-VEN: Chidamide, demethylating drugs (azacitidine), cytarabine, aclacinomycin, G-CSF, and venetoclax. (B) CDCAG: Chidamide, demethylating drugs (decitabine), cytarabine, aclacinomycin, and G-CSF.


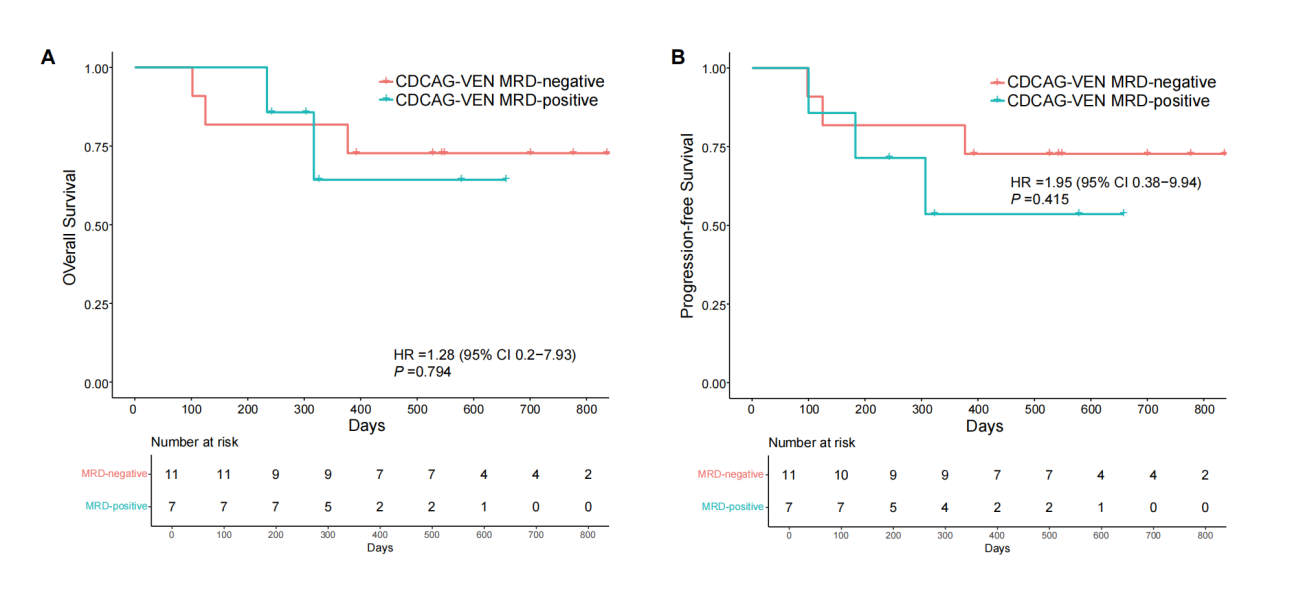


Figure S3. Survival outcomes in CDCAG+VEN- and CDCAG-treated patients. (A) OS in CDCDA-VEN-treated patients with MRD-negative or MRD-positive disease. (B) PFS in CDCDA-VEN-treated patients with MRD-negative or MRD-positive disease. CDCAG-VEN: Chidamide, demethylating drugs (azacitidine), cytarabine, aclacinomycin, G-CSF, and venetoclax; CDCAG: Chidamide, demethylating drugs (decitabine), cytarabine, aclacinomycin, and G-CSF.


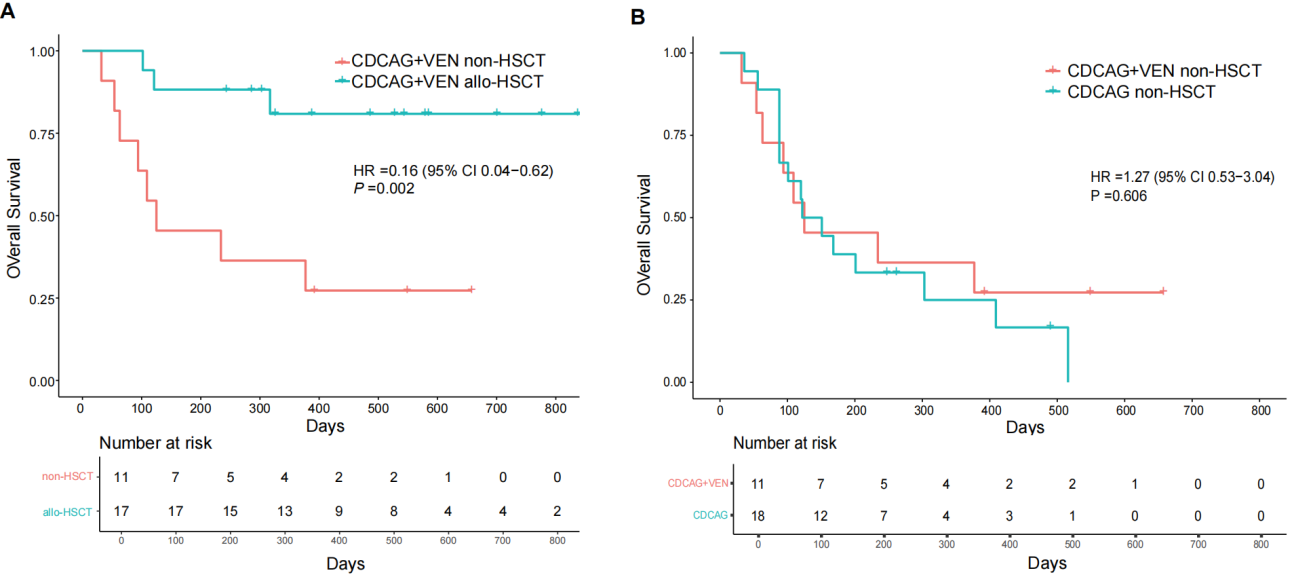


Figure S4. Survival outcomes in CDCAG+VEN- and CDCAG-treated patients. (A) OS in CDCDA-VEN-treated patients with allo-HSCT or non-HSCT. (B) OS in non-HSCT patients with CDCAG-treated or CDCAG-VEN-treated. (CDCAG-VEN: Chidamide, demethylating drugs (azacitidine), cytarabine, aclacinomycin, G-CSF, and venetoclax; CDCAG: Chidamide, demethylating drugs (decitabine), cytarabine, aclacinomycin, and G-CSF; allo-HSCT: Allogeneic hematopoietic stem cell transplantation
