# Supplementary material for: Chidamide in Combination With DCAG With or Without Venetoclax for Relapsed/Refractory Acute Myeloid Leukemia
Source: Cancer Med. 2025 Mar 10;14(5):e70734. doi: 10.1002/cam4.70734 (PMC11891779; doi:10.1002/cam4.70734)
Supplement: Supplementary file 2 — Data S2. [file CAM4-14-e70734-s001.docx]

**Table S1. Details of patient inclusion and exclusion criteria**

| **Patients criteria** |
| --- |
| Voluntary participation in the clinical study |
| The person or legal guardian fully understands and signs the Informed Consent Form (ICF) |
| Willing to follow and be able to complete all trial procedures |
| Conform to the 2022 WHO diagnostic criteria for acute myeloid leukemia, excluding acute promyelocytic leukemia |
| Age: 14–75 years old, regardless of sex |
| No severe allergies |
| Liver function: ALT/AST ≤ 2.5 times normal, bilirubin ≤ 2 times normal |
| Renal function: creatinine ≤ normal |
| There are no uncontrollable infections or serious mental disorders |
| Eastern Cooperative Oncology Group (ECOG) performance status of 0–2, the estimated survival time is greater than or equal to 4 months |

**Table S2. Demographics of patients in the CDCAG-VEN group**

| Pt# | Age (y) | Sex | Disease status | ECOG | FAB | 2023 NCCN risk status | Molecular | Baseline parameters | | | | Prior therapy  lines | Prior therapy regimen |
| --- | --- | --- | --- | --- | --- | --- | --- | --- | --- | --- | --- | --- | --- |
|  |  |  |  |  |  |  |  | WBC | Hb | Plt | BM  blasts (%) |  |  |
| 1 | 74 | M | Refractory | 2 | M2 | Adverse | SRSF2/ASXL1/RUNX1/STAG2/CREBBP | 2.76 | 67 | 10 | 77 | 1 | AZA+CAG(1) |
| 2 | 38 | M | Refractory | 2 | M0 | Intermediate | FLT3-ITD/SETD2/ZBTB7A | 0.83 | 74 | 197 | 89 | 3 | IA/CAG/SKL1028 |
| 3 | 61 | M | Refractory | 2 | M2 | Favorable | DNMT3A/KIT | 1.3 | 71 | 48 | 90 | 1 | VEN+AZA |
| 4 | 70 | M | Refractory | 2 | M4 | Adverse | BCOR/DNMT31/GATA2/NRAS/  ASXL1/NF1 | 3.28 | 68 | 16 | 22 | 1 | VEN+AZA+Ara-c |
| 5 | 43 | M | Refractory | 2 | M5b | Adverse | / | 15.26 | 72 | 23 | 50 | 3 | AZA+HA/AZA+Aclarubicin+HAG/VDAP+Cladribine |
| 6 | 33 | M | Refractory | 1 | M2 | Adverse | TP53/WT1 | 3.54 | 80 | 16 | 48.4 | 3 | IDA/AZA+HD-Ara-c/VEN+AZA |
| 7 | 41 | F | Relapse | 2 | M0 | Adverse | KIT/IDH2/ASXL1 | 17.2 | 57 | 21 | 13 | 15 | IA/IA+Decitabine/HD-Ara-c(2)/AZA+HAG/AZA+CAG+PD1/VEN+PD1/VEN+AZA+Selinexor/FLAG(3)/AZA+CAG+PD1/AZA+VEN+Ara-c/AZA+VEN+Selinexor |
| 8 | 15 | M | Relapse | 2 | M5b | Intermediate | KMT2A/NRAS | 18.41 | 124 | 34 | 62 | 3 | IA+VP16+Luxolitinib/HD-Ara-c+Chidamide/HD-Ara-c |
| 9 | 52 | F | Relapse | 1 | M4 | Favorable | Biallelic CEBPA/TET2 | 2.58 | 94 | 132 | 77 | 4 | Decitabine+IA/HD-Ara-c(3) |
| 10 | 42 | F | Relapse | 1 | M5b | Intermediate | / | 2.58 | 94 | 132 | 62 | 7 | DA/HD-Ara-c(5)/MA |
| 11 | 32 | F | Relapse | 2 | M2 | Intermediate | / | 2.16 | 90 | 65 | 95 | 6 | IA/VEN+AZA+CAG(5) |
| 12 | 59 | F | Relapse | 1 | M4 | Intermediate | FLT3-TKD/STAG2/SRSF2/NRAS/KRAS | 6.28 | 95 | 62 | 35.6 | 3 | VEN+AZA/HVA/HD-Ara-c |
| 13 | 58 | M | Relapse | 1 | M2 | Intermediate | DNMT3A/PHF6 | 2.51 | 81 | 39 | 37 | 7 | IA/AZA(3)/HD-Ara-c(2)/TA |
| 14 | 35 | F | Relapse | 1 | M5 | Intermediate | FLT3-ITD/KIT | 15.18 | 78 | 6 | 59.5 | 2 | IA(2) |
| 15 | 27 | F | Relapse | 1 | M5 | Intermediate | IDH1/NRAS | 2.5 | 110 | 154 | 45 | 6 | DA(2)/HD-Ara-c(3)/AA |
| 16 | 19 | M | Relapse | 1 | M5 | Adverse | FLT3-ITD/TET2/TP53 | 5.68 | 69 | 30 | 64 | 2 | IA/HD-Ara-c |
| 17 | 31 | F | Relapse | 2 | M4 | Intermediate | DNMT3A/FLT3-TKD/GATA2/IDH1/MYC | 2.36 | 96 | 77 | 29.6 | 2 | IA/DCIG |
| 18 | 46 | M | Refractory | 2 | M4 | Intermediate | / | 0.63 | 74 | 114 | 78.4 | 1 | IA |
| 19 | 33 | F | Refractory | 2 | M4 | Favorable | NRAS/DNMT3/IDH1/NPM1/KRAS | 4.02 | 94 | 316 | 76 | 2 | IA/AZA+VEN+Chidamide |
| 20 | 39 | F | Relapse | 1 | M2 | Adverse | Biallelic-CEBPA/ASXL1 | 4.38 | 115 | 64 | 40.7 | 7 | IA(2)/IAE10/HD-Ara-c(4) |
| 21 | 45 | F | Refractory | 1 | M5 | Adverse | FLT3-ITD/RUNX1/KMT2A/DDX41 | 1.63 | 73 | 42 | 87.2 | 1 | DDAG |
| 22 | 30 | M | Refractory | 1 | M2 | Intermediate | / | 1.28 | 52 | 9 | 20 | 17 | CAG(2)/TA(6)/IA(2)/Decitabine+HAA(3)/Decitabine+CAELYX+HA(2)/AZA+VP-16+HA/IDA+HD-Ara-c |
| 23 | 65 | M | Refractory | 2 | / | Intermediate | / | 2.49 | 56 | 143 | 28 | 1 | VEN+Alarubicin+Ara-c |
| 24 | 16 | M | Refractory | 1 | M4 | Adverse | KRAS/BCOR | 1.54 | 71 | 16 | 55.5 | 1 | DA |
| 25 | 57 | M | Refractory | 2 | M2/M5 | Intermediate | / | 2.81 | 45 | 92 | 26.5 | 1 | HAA+Ara-c |
| 26 | 59 | M | Refractory | 2 | / | Adverse | TP53 | 2.91 | 62 | 36 | 25 | 1 | AZA+VEN+Decitabine |
| 27 | 54 | F | Refractory | 1 | M5b | Adverse | TP53 | 30.17 | 71 | 21 | 63 | 2 | DA(1)/Lenalidomide+VEN |
| 28 | 46 | F | Relapse | 1 | / | Intermediate | IDH1/IDH2/WT1 | 0.97 | 72 | 12 | 18 | 4 | IA(2)/AZA+VEN/DA |
| 29 | 67 | M | Relapse | 1 | / | Intermediate | / | 142.82 | 56 | 41 | 56 | 4 | AZA+VEN(2)/AZA/AZA+Ara-c |
| 30 | 58 | F | Refractory | 1 | M2a | Adverse | IDH2/TET2/BCOR/CREBBP/DNMT3A/IGLL5 | 1.3 | 81 | 90 | 45.5 | 1 | DA |

CDCAG-VEN: Chidamide, demethylating drugs (azacitidine), cytarabine, aclacinomycin, G-CSF, and venetoclax.

**Table S3. Demographics of patients in the CDCAG group**

| Pt# | Age (y) | Sex | Disease status | ECOG | FAB | 2023 NCCN risk status | Molecular | Baseline parameters | | | | Prior therapy  lines | Prior therapy regimen |
| --- | --- | --- | --- | --- | --- | --- | --- | --- | --- | --- | --- | --- | --- |
|  |  |  |  |  |  |  |  | WBC | Hb | Plt | BM  blasts (%) |  |  |
| 1 | 71 | F | Refractory | 2 | M5 | Intermediate | NRAS/IDH2/BCOR | 0.57 | 81 | 27 | 88 | 1 | DCAG |
| 2 | 53 | M | Refractory | 2 | M5 | Adverse | FLT3-ITD | 158.31 | 96 | 25 | 94 | 1 | DCAG+SOR |
| 3 | 29 | M | Refractory | 0 | M5 | Intermediate | U2AF1/IDH1/RUNX1 | 1.65 | 92 | 36 | 53.2 | 1 | IA |
| 4 | 12 | M | Refractory | 1 | M2 | Intermediate | FLT3-ITD/RUNX1 | 7.84 | 59.4 | 48 | 66 | 2 | DA/DCAG |
| 5 | 55 | M | Refractory | 0 | M5 | Intermediate | KRAS/DNMT3A | 31.84 | 49 | 220 | 83.6 | 2 | MA/DCAG |
| 6 | 65 | F | Relapse | 1 | M5 | Intermediate | CEBPA | 137.63 | 81 | 51 | 83.6 | 3 | DCAG(3) |
| 7 | 29 | F | Refractory | 1 | M5 | Intermediate | / | 49.9 | 111 | 109 | 76.5 | 1 | IA |
| 8 | 57 | M | Relapse | 2 | M5 | Intermediate | FLT-ITD/DNMT3A/NPM1 | 112 | / | / | 89 | 5 | IA+SOR/HD-Ara-c(3)/HAA |
| 9 | 17 | M | Refractory | 2 | M4 | Intermediate | IDH2/NRAS/WT1/BCOR | 28 | 37 | 113 | 50 | 1 | MAE |
| 10 | 51 | F | Refractory | 0 | M4 | Intermediate | JAK2 | 15.49 | 134 | 621 | 47.2 | 2 | MAE/Chidamide+MAE |
| 11 | 24 | M | Relapse | 2 | M5a | Adverse | IDH1/NOTCH1/KMT2D/CRLF2 | 14.15 | 114 | 27 | 94.5 | 4 | IA/HD-Ara-c/CAG/DCAG |
| 12 | 39 | F | Refractory | 0 | M4/M5 | Intermediate | NPM1/DNMT3A/FLT3-ITD | 89.78 | 86 | 18 | 54 | 1 | MA |
| 13 | 58 | F | Relapse | 0 | M4 | Adverse | / | 25.93 | 61 | 51 | 46.4 | 16 | DA(4)/HD-Ara-C(5)/HA/MA(3)/CAG/MAE/Chidamide |
| 14 | 29 | M | Refractory | 0 | M2 | Intermediate | NOTCH1/SETBP1/TNFAIP3 | 311 | 76 | 24 | 85.2 | 4 | DA/CAG(2)/MAC |
| 15 | 65 | F | Refractory | 2 | M4 | Intermediate | WT1/NPM1/FLT3-ITD | 145.35 | 70 | 57 | 80 | 2 | IA/DCAG+SOR |
| 16 | 28 | F | Refractory | 1 | M5 | Intermediate | TET2/NPM1/FLT3/DNMT3A/WT1 | 49.13 | 79 | 226 | 87 | 1 | MA |
| 17 | 48 | M | Relapse | 1 | M5 | Intermediate | / | / | / | / | / | 16 | / |
| 18 | 15 | M | Refractory | 2 | M2 | Favorable | IDH1/NRAS/CEBPA/WT1 | 32.28 | 74 | 7 | 85.2 | 1 | IA |
| 19 | 34 | M | Refractory | 2 | M5 | Intermediate | NPM1/FLT3-ITD | 116.8 | 95 | 29 | 77.2 | 3 | IA(2)/FLAG |
| 20 | 51 | F | Relapse | 1 | M4 | Favorable | / | 14.25 | 116 | 35 | 78 | 7 | MA/DCAG/HD-Ara-C(4)/DCAG |
| 21 | 48 | M | Refractory | 0 | / | Intermediate | / | 4.26 | 101 | / | / | 1 | IA |
| 22 | 15 | M | Relapse | 2 | M2 | Favorable | / | 32.9 | 83 | 72 | 44.8 | 4 | MA/HD-Ara-C(2)/DCAG |

CDCAG: Chidamide, demethylating drugs (decitabine), cytarabine, aclacinomycin, and G-CSF.

**Table S4. Treatment response after one cycle of CDCAG-VEN and CDCAG treatment by 2023 NCCN risk status**

| Outcomes | **CDCAG-VEN (n = 28)** | | | **CDCAG (n = 22)** | | |
| --- | --- | --- | --- | --- | --- | --- |
|  | Favorable  (n = 3) | Intermediate  (n = 15) | Adverse  (n = 10) | Favorable  (n = 3) | Intermediate  (n = 16) | Adverse  (n = 3) |
| ORR, n% (95% CI) | 100 (31.0–100) | 80.0 (51.4–94.70) | 70.0 (35.4–91.9) | 66.7 (12.5–98.2) | 43.8 (20.1–69.4) | 33.3 (1.8–87.5) |
| CRc, n% (95% CI) | 100 (31.0–100) | 66.7 (38.7–87.0) | 50.0 (20.1–79.9) | 33.3 (1.8–87.5) | 37.5 (16.3–64.1) | 33.3 (1.8–87.5) |
| MRD^-^CRc, n% (95% CI) | 66.7 (12.5–98.2) | 70.0 (35.3–91.9) | 40.0 (7.3–83.0) | 0 | 33.3 (6.0–75.9) | 0 |
| PR, n% (95% CI) | 0 | 13.3 (2.3–41.6) | 20.0 (3.5–55.8) | 0 | 6.3 (0.3–32.2) | 0 |
| NR, n% (95% CI) | 0 | 20.0 (5.3–48.6) | 30.0 (8.1–64.6) | 33.3 (1.8–87.5) | 56.3 (30.6–79.3) | 33.3 (1.8–87.5) |

CDCAG-VEN: Chidamide, demethylating drugs (azacitidine), cytarabine, aclacinomycin, G-CSF, and venetoclax; CDCAG: Chidamide, demethylating drugs (decitabine), cytarabine, aclacinomycin, and G-CSF; CI: Confidence interval; ORR: Overall response rate; CRc: Composite complete remission; MRD: Measurable residual disease; PR: Partial remission; NR: No response.

**Table S5: Survival outcomes in CDCAG-VEN- and CDCAG-treated patients**

|  | **CDCAG-VEN (n = 28)** | **CDCAG (n = 28)** | **HR (95% CI)** | ***P*** |
| --- | --- | --- | --- | --- |
| Follow up  Median, days (95% CI) | 544 (243–917) | 490 (248–2203) |  |  |
| Overall survival  Median, days (95% CI)  6-month OS  12-month OS | NA (234–NA)  71.4%  63.6% | 184.5, (88–369)  50.0%  35.1% | 0.39 (0.19–0.79) | 0.005 |
| Progression-free survival  Median, days (95% CI)  6-month PFS  12-month PFS | NA (377–NA)  86.4%  76.7% | 318, (57–NA)  70.0%  36.0% | 0.30 (0.10–0.90) | 0.022 |
| Duration of response  Median, days (95% CI)  6-month DOR  12-month DOR | NA (344–NA)  81.8%  71.2% | 265, (34–NA)  80.0%  34.3% | 0.09 (0.10–0.89) | 0.021 |
| Cumulative incidence of relapse  Median, days (95% CI)  6-month CIR  12-month CIR | NA (NA–NA)  11.1%  22.2% | 518, (164–NA)  22.6%  48.9% | 0.35 (0.10–1.20) | 0.095 |

CDCAG-VEN: Chidamide, demethylating drugs (azacitidine), cytarabine, aclacinomycin, G-CSF, and venetoclax; CDCAG: Chidamide, demethylating drugs (decitabine), cytarabine, aclacinomycin, and G-CSF.

**Table S6.** **Hematological recovery in CDCAG-VEN- and CDCAG-treated patients**

|  | CDCAG-VEN | CDCAG | *P* |
| --- | --- | --- | --- |
| Days for neutrophils  > 0.5 × 10^9^ /L | 18.0 (11.0–26.0) | 19.0 (12.0–26.0) | 0.293 |
| Days for platelets  > 20 × 10^9^ /L | 18.0 (10.0–26.0) | 19 (13.0–26.0) | 0.311 |
| Packed red cells units (range) | 9.5 (0–26.3) | 10.2 (1.0–28.2) | 0.282 |
| Platelet units (range) | 9.2 (1.5–23.0) | 10.0 (1.0–24.0) | 0.322 |

CDCAG-VEN: Chidamide, demethylating drugs (azacitidine), cytarabine, aclacinomycin, G-CSF, and venetoclax; CDCAG: Chidamide, demethylating drugs (decitabine), cytarabine, aclacinomycin, and G-CSF.
